# Surgical technique of spine-shortening vertebral osteotomy for adult tethered cord syndrome: a case report and review of the literature

**DOI:** 10.1186/s13256-023-04155-x

**Published:** 2023-10-11

**Authors:** Takashi Kobayashi, Naohisa Miyakoshi, Toshiki Abe, Kazuma Kikuchi, Eiji Abe, Yoichi Shimada

**Affiliations:** 1 Department of Orthopedic Surgery, Akita Kousei Medical Center, 1-1-1 Iijima, Nishifukuro, Akita, 011-0948 Japan; 2 Department of Orthopedic Surgery, Akita University Graduate School of Medicine, 1-1-1 Hondo, Akita, 010-8543 Japan

**Keywords:** Case report, Spine-shortening vertebral osteotomy, Adult tethered cord syndrome, Surgical technique

## Abstract

**Background:**

Miyakoshi *et al.* reported three cases of tethered cord syndrome treated by spine-shortening vertebral osteotomy, which provided relief of the patients’ symptoms with no complications. Although the details of these cases were described in a previous report, the surgical technique was not thoroughly explained. In the present report, we describe the details of our procedure with reference to a fourth case.

**Case presentation:**

A 47-year-old Asian woman was admitted to our hospital with a 1-year history of worsening leg numbness and urinary dysfunction. Magnetic resonance imaging revealed a low-lying conus medullaris extending to the level of S2 and surrounded by fat tissue at that level. We diagnosed her condition as adult tethered cord syndrome, and spine-shortening vertebral osteotomy was planned. The target level for the osteotomy was L2. Bilateral pedicle screw implants were placed at L1 and L3 using an anterior–posterior image intensifier. In this procedure, it is essential to use monoaxial screws inserted exactly parallel to the rostral endplates of each vertebral body; this ensures appropriate alignment between the L1 caudal endplate and the L2 osteotomy surface. The upper one-third of the lamina of L2 was resected, and the bilateral two-thirds of the pedicle of L2 was removed with a surgical air drill. After exposure of the lateral side of the L1–2 disc, discectomy was performed with a knife and curette. Following complete discectomy of L1–2, the upper vertebral body of L2 was removed with a surgical air drill. After complete removal of the vertebral body, a straight rod was connected to two screws and applied pressure between the screws. Two polyethylene tapes were applied to the L2 lamina and bilateral rods.

**Conclusion:**

Spine-shortening osteotomy that preserves the caudal one-third of the pedicle and lamina with one-above and one-below instrumentation successfully reduced the spinal cord tension without causing neural damage.

## Background

Miyakoshi *et al.* [1] described the first case series of spine-shortening vertebral osteotomy for tethered cord syndrome in the English-language literature. They reported three cases of tethered cord syndrome treated by spine-shortening vertebral osteotomy, which provided relief of the patients’ symptoms with no complications. Although Miyakoshi *et al.* [1] described the details of these cases, they did not thoroughly explain the surgical technique. In the present report, we describe the details of our procedure with reference to a fourth case.

## Case presentation

**Fig. 1:**
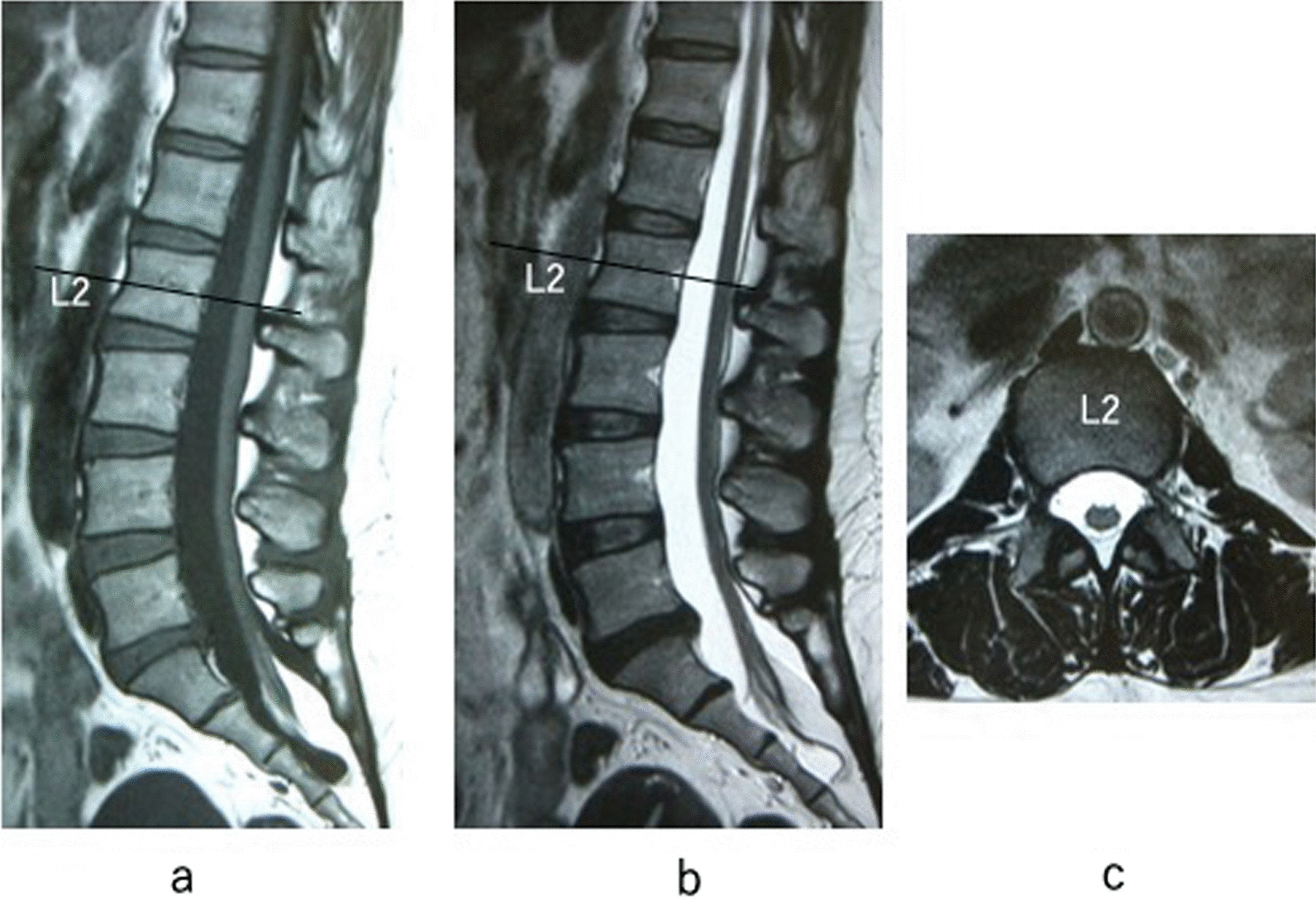
Preoperative lumbar magnetic resonance images of a 47-year-old woman. Magnetic resonance imaging revealed a low-lying conus medullaris extending to the level of S2 and surrounded by fat tissue at that level. **a** T1 sagittal image. **b** T2 sagittal image. **c** T2 axial image

A 47-year-old Asian woman was admitted to our hospital with a 1-year history of worsening leg numbness and urinary dysfunction. She first noticed her bilateral 3rd and 4th toe numbness for 5 years. Her symptoms worsened, and her whole toes became numb a year ago. She had also been experiencing frequent urination for a year. She visited her previous physician and was referred to our hospital after magnetic resonance imaging was taken. On physical examination, her motor function was intact, but she had sensory disturbance around the anal region. Deep tendon reflexes were normal. Her finger–floor distance was 10 cm, and her straight leg raise test result was 80 degrees; this indicated that her hamstrings were not tight. Magnetic resonance imaging revealed a low-lying conus medullaris extending to the level of S2 and surrounded by fat tissue at that level (Fig. 1). The spinal MRI did not point to any other abnormalities that could explain her symptoms. We diagnosed her condition as adult tethered cord syndrome, and spine-shortening vertebral osteotomy was planned. After the patient provided informed consent, she underwent shortening osteotomy.

### Surgical procedure

**Fig. 2:**
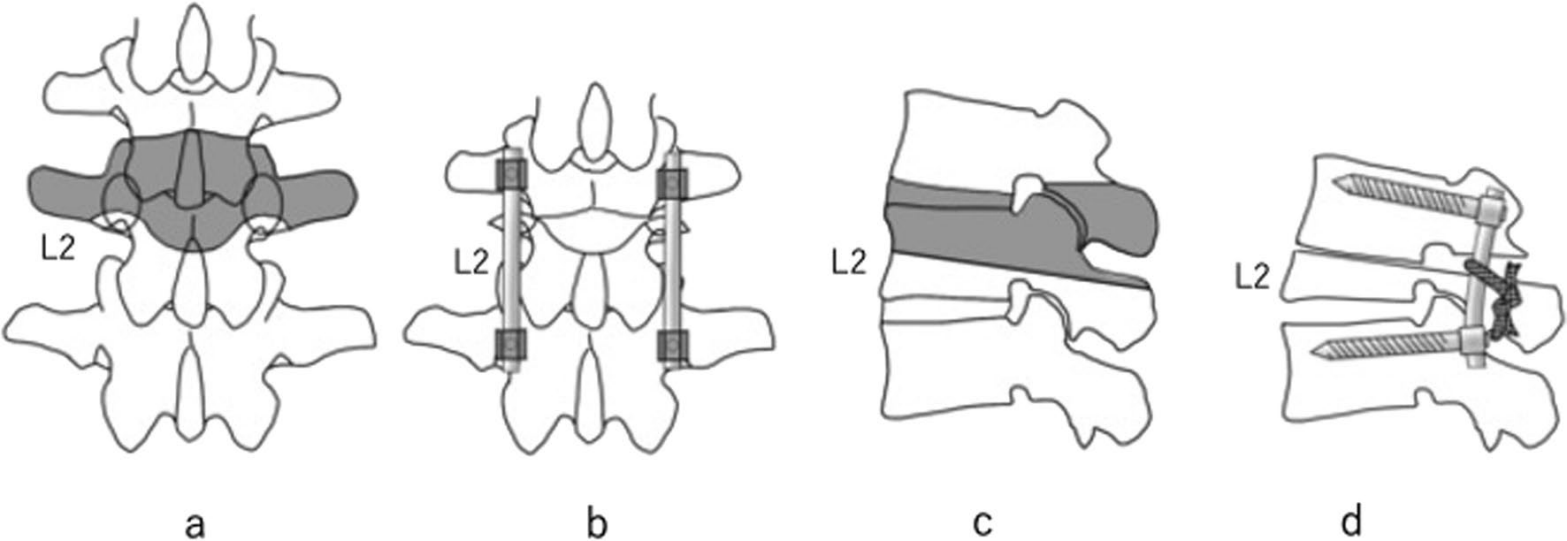
A drawing of the surgical procedure of shortening osteotomy. **a** Posterior image. The black shadow shows the area of the osteotomy. **b** Postoperative image. **c** Lateral image. The black shadow shows the area of the osteotomy. **d** Postoperative lateral image. During this procedure, it is essential to use monoaxial screws inserted exactly parallel to the rostral endplates of the vertebral bodies. Taping to the L2 lamina and bilateral rods makes the osteotomy site biomechanically stable

After induction of general anesthesia, the patient was positioned onto a Jackson Spinal Table (Mizuho Co. Ltd., Tokyo, Japan). Neurophysiological monitoring was performed using motor evoked potentials. A midline incision was made from the T11 to L4 spinous process level. The target level for the osteotomy was L2. The L2 segment was extensively exposed, in turn exposing the posterior element and transverse processes bilaterally. Bilateral pedicle screw implants were placed at L1 and L3 using an anterior–posterior image intensifier. In this procedure, it is essential to use monoaxial screws inserted exactly parallel to the rostral endplates of each vertebral body; this ensures appropriate alignment between the L1 caudal endplate and the L2 osteotomy surface. The osteotomy was started after insertion of the pedicle screws. First, the lower half of the L1 lamina and bilateral inferior articular processes of L1 as well as the bilateral L2 superior articular processes were resected. Second, the upper one-third of the lamina of L2 was resected, and the bilateral two-thirds of the pedicle of L2 was removed with a surgical air drill. Resection of the upper one-third of the lamina of L2 is very important to prevent postoperative neurological deterioration due to epidural hematoma formation. Although bone union can effectively occur without resection of the lamina, the dural space will be so tight that only a small hematoma will be symptomatic. After exposure of the lateral side of the L1–2 disc, discectomy was performed with a knife and curette. Following complete discectomy of L1–2, the upper vertebral body of L2 was removed with a surgical air drill. The surgical air drill was inserted from the pedicle parallel to the upper endplate of L2, and the posterior wall of the vertebral body was thus removed. After thinning of the lateral vertebral cortex, the lateral surface of the vertebral body was carefully exposed, and the lateral cortex was removed with a punch. After thinning of the anterior vertebral cortex, a Kerrison rongeur was used to remove the anterior cortex. Because the anterior longitudinal ligament protects the vessels and anterior organs, little bleeding occurred when the anterior body was removed. After complete removal of the vertebral body, a straight rod was connected to two screws and applied pressure between the screws. Two polyethylene tapes (Alfresa Pharma Corporation, Osaka, Japan) were applied to the L2 lamina and bilateral rods. A drawing of the surgery is shown in Fig. 2. The operation time was 5 h 13 min, and the estimated blood loss was 108 ml. In this case, there was less degeneration, and the epidural venous plexus was less developed, which may have been the reason for the small amount of blood loss.

The tips and tricks of this procedure are as follows. (1) Carefully develop the sides of the vertebral body and intervertebral disc with hemostasis; (2) use the endplate of the vertebral body as a guide and insert an air tome parallel to the endplate to resect the vertebral body; (3) leave the lateral wall of the vertebral body like an eggshell and complete the resection by resecting the shell at the end.

### Postoperative course

**Fig. 3:**
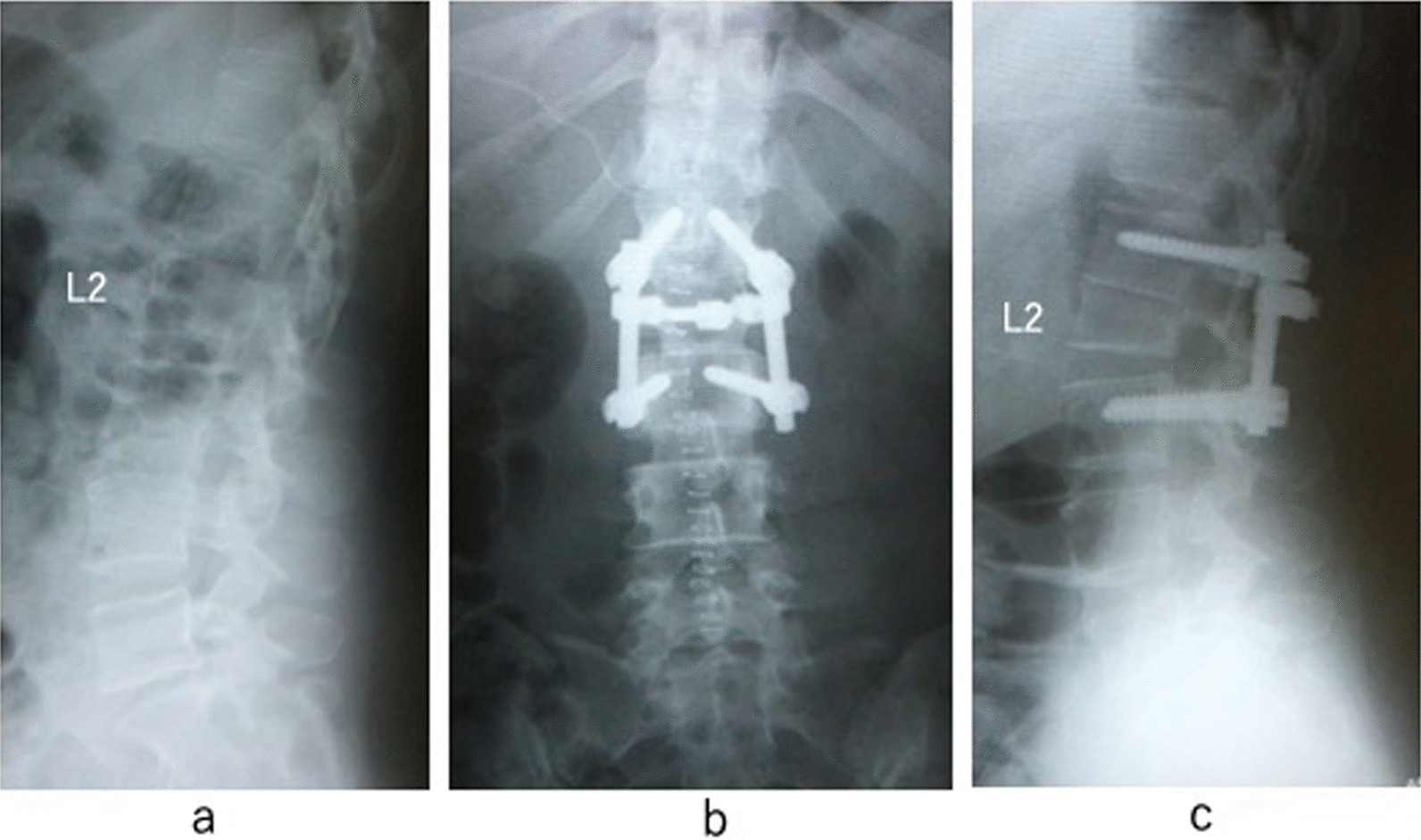
Preoperative and postoperative radiographs. **a** Preoperative lateral radiograph. **b** Postoperative anteroposterior radiograph. **c** The postoperative lateral radiograph shows 18-mm shortening of the spine compared with the preoperative radiograph

**Fig. 4:**
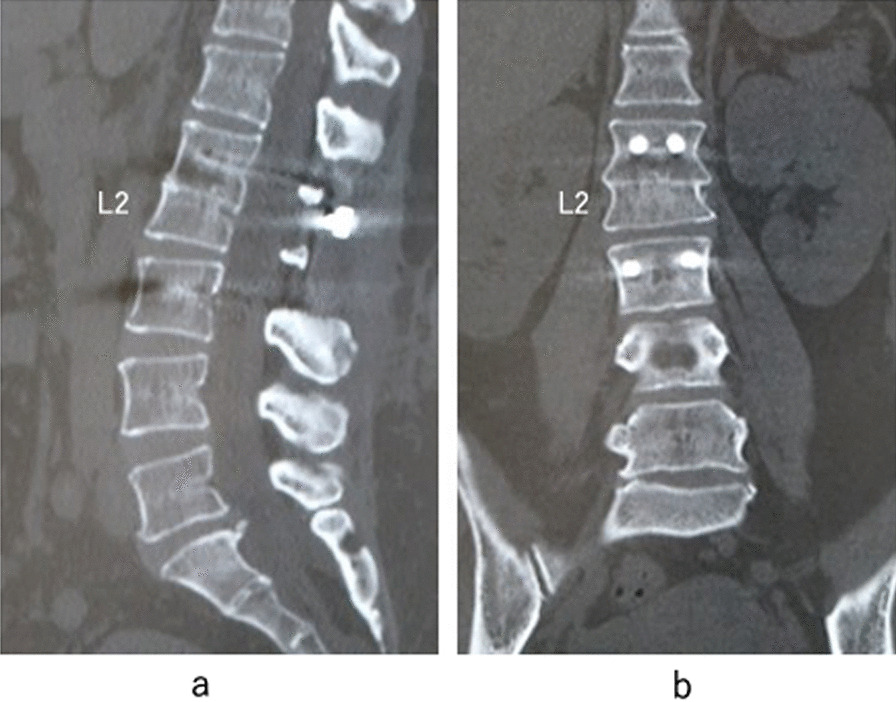
One-year postoperative computed tomography images. **a** Sagittal reconstruction image. **b** Coronal reconstruction image. Complete bone union was achieved

The postoperative course was uneventful. The postoperative radiograph showed 18-mm shortening from the L1 upper endplate to the L2 lower endplate (Fig. 3). The patient’s leg numbness improved immediately after surgery, and her urinary disturbance improved 1 year after surgery, although magnetic resonance imaging did not show evidence of untethering. Computed tomography 1 year after the operation showed complete bone union (Fig. 4); therefore, we removed the instrumentation. She had developed no recurrence at 2 years after surgery.

## Discussion and conclusion

Tethered cord syndrome is a disorder involving abnormal stretching of the tethered spinal cord and is caused by several pathological conditions. Surgery must be performed as soon as possible to improve the symptoms [2, 3]. Although untethering is the gold standard treatment for tethered cord syndrome, it is technically challenging to accomplish without inducing neurological complications [4, 5]. Furthermore, the recurrence rate of untethering ranges from 5 to 50% [6].

Spine-shortening vertebral osteotomy was first proposed by Kokubun [7] to indirectly minimize spinal cord tension. Kanno *et al.* [8] described a 57-year-old woman with tethered cord syndrome complicated by a T12 vertebral fracture that was successfully treated by spine-shortening vertebral osteotomy of T12 without complications. Miyakoshi *et al.* [1] reported the first case series of this novel procedure without complications in the English-language literature. Kokubun *et al.* [9] reported their original method involving eight patients. Hsieh *et al.* [10] described the surgical technique of posterior vertebral column subtraction for the treatment of multiple recurrences of tethered cord syndrome. According to a cadaveric tethered cord model, shortening the vertebral column by 15 to 25 mm significantly reduced spinal cord, lumbosacral nerve root, and filum terminale tension [11].

This procedure indirectly reduces the tension in the spinal cord and is a valuable technique that can be applied to both initial and revision surgery cases. Since this procedure involves only the vertebral body, it is relatively easy to master for spine surgeons who have mastered basic spine techniques.

We chose L2 as the target vertebra for shortening osteotomy because this vertebra has several advantages over the thoracic vertebrae. First, because no ribs are present at L2, shortening is easier than for the thoracic vertebra. Second, osteotomy performed at a lower level will result in less severe neuropathy, if any. Kokubun *et al.* [9] reported a case of severe neurological deterioration postoperatively. The cause of this unexpected result was unknown, but severe adhesion was present between the arachnoid and cord at the osteotomy level at the time of the reoperation. Third, L3 can serve as the distal fusion level. More distal fusion level increases the risk of distal junction failure [12]. Whether L3 is a safe level for distal fusion remains unclear; however, our patient did not develop distal junction failure throughout a 2-year follow-up.

Although instrumentation involving two levels rostral and caudal to the target vertebra has been recommended [10], we chose short fusion from one level rostral to one level caudal because short fusion is ideal if the stability is satisfactory. Because we preserved the caudal one-third of the pedicle and lamina, we were able to augment the lamina and rod construct with polyethylene tape. Although the caudal one-third of the pedicle remained, the length of the shortening was 18 mm, which was enough to reduce the spinal cord tension [11].

This procedure has an important limitation. If the pedicle of the target vertebra is so small that inadequate shortening is expected, total removal of the pedicle will be needed, and posterior vertebral column subtraction osteotomy should be chosen in such cases [10].

In conclusion, spine-shortening osteotomy that preserves the caudal one-third of the pedicle and lamina with one-above and one-below instrumentation successfully reduced the spinal cord tension without causing neural damage.

## Data Availability

The datasets used during the current study are available from the corresponding author on reasonable request.
